# Bioactive Compounds, Antioxidant Activity and Sensory Properties of Northern Red Oak (*Quercus rubra* L., syn. *Q. borealis* F. Michx) Seeds Affected by Roasting Conditions

**DOI:** 10.3390/molecules28052299

**Published:** 2023-03-01

**Authors:** Joanna Oracz, Monika Prejzner, Joanna Grzelczyk, Gabriela Kowalska, Dorota Żyżelewicz

**Affiliations:** Institute of Food Technology and Analysis, Faculty of Biotechnology and Food Sciences, Lodz University of Technology, 2/22 Stefanowskiego Street, 90-537 Lodz, Poland

**Keywords:** *Quercus rubra* L., roasting, phenolic compounds, melanoidins, antioxidant properties, color, electronic tongue

## Abstract

The nutritional value and health-promoting properties cause the fruits (acorns) of *Quercus* spp. to have great potential for use in the food industry as functional ingredients and antioxidants source. The aim of this study was to examine the bioactive compound’s composition, antioxidant potential, physicochemical properties and taste characteristics of northern red oak (*Quercus rubra* L.) seeds subjected to roasting at different temperatures and times. The results indicate that the roasting markedly affects the composition of bioactive components of acorns. In general, the use of roasting temperatures greater than 135 °C causes a decrease in the total phenolic compound content of *Q. rubra* seeds. Furthermore, along with an increase in temperature and thermal processing time, a remarkable increase in melanoidins, which are the final products of the Maillard reaction, was also observed in processed *Q. rubra* seeds. Both unroasted and roasted acorn seeds had high DPPH radical scavenging capacity, ferric reducing antioxidant power (FRAP) and ferrous ion chelating activity. Roasting at 135 °C caused negligible changes in total phenolics content and antioxidant activity of *Q. rubra* seeds. Almost all samples had lower antioxidant capacity along with an increase in the roasting temperatures. Additionally, thermal processing of acorn seeds contributes to the development of the brown color and the reduction of bitterness, and the creation of a more pleasant taste of the final products. Overall, the results of this study show that both unroasted and roasted *Q. rubra* seeds may be an interesting source of bioactive compounds with high antioxidant activity. Therefore, they can be used as a functional ingredient of beverages or food.

## 1. Introduction

The growing knowledge of the health–diet relationship and understanding that diet is a vital part of maintaining good health lead to the development of functional foods from a variety of natural sources, such as fruits, vegetables, nuts and seeds. A healthy and varied diet rich in all essential ingredients should not only be of interest to nutritionists, but, due to the wide availability of highly processed foods, it is becoming a political issue due to its potentially positive impact on climate change, the health crisis and food shortage.

The fruits (acorns) of *Quercus* spp. are a promising source of bioactive compounds and nutrients for human diet and functional food applications [1]. In recent years, there has been an increasing interest in using acorns as new ingredients for bread and pastry production and as cocoa or coffee substitutes, since their composition is comparable to that of cereal grains [2,3,4,5]. The acorn seeds contain considerable amounts of nutrients, such as carbohydrates, mainly starch (31–51%), proteins (2–8%), fats (0.7–9%) and also minerals, essential amino acids, vitamins, unsaturated fatty acids, tocopherols and sterols [1,3,6,7,8]. Moreover, some studies have also shown that the seeds of *Quercus robur* (English oak), *Quercus faginea* (Portuguese oak), *Quercus ilex* (Holm oak), *Quercus cerris* (Turkish oak) and *Quercus rubra* (northern red oak) are a rich source of phenolic compounds, including hydrolysable tannins (gallotannins and ellagitannins), phenolic acids (gallic and ellagic acids and their derivatives) and flavonoids (flavan-3-ols and flavonols) [1,2,9,10] with confirmed pharmacological properties [1,10,11,12,13]. The biological activities of phenolic compounds found in acorn seeds are related to their antioxidant, anti-inflammatory, anticancer, antimicrobial, neuroprotective and cardioprotective properties [1]. The high consumption of these compounds may reduce the risk of cardiovascular and inflammatory diseases, cancers, diabetes, microbial infection and other age-related disorders [1,11,13,14].

Currently, the acceptance and consumption of products derived from acorn seeds are still limited by their pronounced bitterness and astringency. It is well known that phenolic compounds play an important role not only in the health-promoting effects of plant materials and foods but also in their sensory properties [1,2,15]. The bitter taste of *Quercus* seeds is mainly related to their high tannin content. The high content of these compounds is responsible for the strong bitter taste of acorn seeds and their derived products, including flour and coffee or cocoa substitutes [1]. Thus, fresh acorn seeds are not commonly consumed directly, but usually after thermal processing, including roasting, boiling or frying [3]. Roasting is one of the most widely used methods for development and enhancement of the sensory quality attributes of acorn seeds, including texture, aroma, taste and color [1]. The chemical reactions that occur during thermal processing of the seeds or nuts are mainly caramelization, Maillard reactions and lipid oxidation [16]. Due to the complexity of these reactions, the heat-treated products exhibit different chemical composition and organoleptic characteristics depending on processing conditions applied. Moreover, during thermal processing, phenolic compounds can undergo decomposition and transformation, and create other interactions, such as hydrogen-bonding and ionic and hydrophobic interactions, with components of the food matrix. Degradation and transformation of phenolic compounds, as well as the formation of new compounds through the Maillard reactions, may also lead to changes in the biological properties of the final products [17,18].

So far, only a few studies on the effects of thermal processing on the total and individual phenolic compounds, antioxidant activity and organoleptic properties of seeds (kernels) of different *Quercus* species have been reported [2,3,5]. Previous studies have shown that heat treatment of acorn kernels, generally, leads to a significant reduction in total tannins content, but increases the level of non-tannin phenolics, including gallic acid or ellagic acid [2,5]. The research also revealed that it is possible to modify the total phenolic content and antioxidant activity of the final products in different ways through the thermal processing. The wide diversity of *Quercus* species and other factors including environmental conditions, hybridization among different oak genotypes, masting variability, maturity period and harvesting time affect the content and profile of phytochemicals in acorn seeds [7,8,19,20]. Due to these variations, the processing of different acorns may also result in differences in their health benefits. Thus, the application of thermal processing methods as a tool to improve the organoleptic properties and maintain high biological activity of the products derived from acorn seeds is still a growing area of research [2,3,5].

The northern red oak belongs to the second most widely distributed red oak group in Poland. Although acorn seeds of *Q. rubra* species are characterized by the high content of natural antioxidants such as phenolic compounds and tocopherols, they are rather poorly used as a food ingredient mainly due to their outstanding bitter taste compared to other species of genus *Quercus* [7,10]. Furthermore, to our knowledge, information on the bioactive compounds, antioxidant activity and organoleptic properties of acorn seeds of this species subjected to different roasting conditions is still scarce.

Therefore, the aim of the present work was to investigate the composition of bioactive compounds, including phenolic compounds and Maillard reaction products, antioxidant capacities and taste characteristics of *Q. rubra* seeds as affected by different combinations of temperature and time of roasting to identify suitable and effective thermal processing conditions, which improve the sensory characteristics of the product and maintain or enhance the content of health beneficial bioactive compounds and antioxidant properties.

## 2. Results and Discussion

### 2.1. Effect of Roasting Conditions on Bioactive Compounds Profile in Q. rubra Samples

#### 2.1.1. Effect of Roasting Conditions on Phenolic Compounds

Phenolic compounds are one of the main health promoting substances in acorn fruits. Therefore, evaluating the effect of different roasting conditions on the concentrations of these compounds is very important and, in the case of the food industry, can be very helpful in the development of new food products with a high content of bioactive compounds, strong antioxidant properties and desirable quality characteristics.

**Figure 1:**
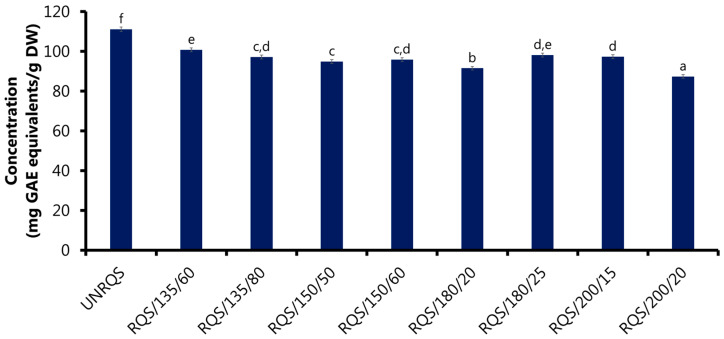
The total phenolics content (Folin–Ciocalteu method) of unroasted and roasted *Q. rubra* seeds. Results are shown as the mean ± standard deviation of three replications (*n* = 3). Bars that share the same superscript letter (a–f) are not significantly different from each other (Tukey’s HSD test, *p* < 0.05); UNRQS, the unroasted seeds; RQS/135/60, seeds roasted at 135 °C/60 min, RQS/135/80, seeds roasted at 135 °C/80 min; RQS/150/50, seeds roasted at 150 °C/50 min; RQS/150/60, seeds roasted at 150 °C/60 min; RQS/180/20, seeds roasted at 180 °C/20 min; RQS/180/25, seeds roasted at 180 °C/25 min; RQS/200/15, seeds roasted at 200 °C/15 min; RQS/200/20, seeds roasted at 200 °C/20 min.

Changes in the total content of phenolic compounds in *Q. rubra* seeds under roasting, at different temperatures and roasting times, are shown in Figure 1. The total phenolics content of *Q. rubra* samples was determined using Folin–Ciocalteu reagent and the results were expressed as milligrams of gallic acid equivalents (GAE) per gram dry weight of sample (mg GAE/g DW). A slight decrease in the total phenolics content was observed in *Q. rubra* samples roasted at 135–200 °C. In general, the unroasted seeds showed higher total phenolics content (111.12 mg GAE/g DW) than roasted samples (87.38–100.82 mg GAE/g DW). The greatest decrease in the total phenolics content was observed when seeds were roasted at 200 °C for 20 min (by 21% of the initial value), while the smallest drop occurred after roasting of seeds at 135 °C for 60 min (by 9% of the initial value).

These observations are in agreement with the results previously obtained by Rakić et al. (2006) [13], who reported that untreated *Q. robur* kernels exhibited higher total phenolics content determined with the Folin–Ciocalteu reagent than samples thermally treated at 200 °C for 20 min. Otherwise, Marc et al. (2021) [3] showed that the total content of phenolics compounds estimated with the Folin–Ciocalteu reagent in the aqueous extracts of *Q. rubra* acorn kernels subjected to roasting at different temperatures increased compared to untreated samples.

In turn, Coelho et al. (2018) [5] found that, depending on the species of *Q. ilex* and *Q. suber* acorns roasted at 225–230 °C for 15 min, the total phenolics content increased or decreased. However, it can be noted that the amount and profile of phytochemicals in *Quercus* spp. fruits differ remarkably between genotypes. Generally, hydrolysable tannins, ellagic acid, gallic acid derivatives and flavonoids are the most abundant phenolic compounds in oak fruits [1,2,6,9,21]. Moreover, such a high variation in the composition of phenolic compounds of different *Quercus* species may be due not only to their genotype, but also to other factors, such as hybridization between different oak species, masting behavior, environmental conditions, maturity stage and harvest time [7,8,20].

The present study is limited only to the results of the total phenolics content of *Q. rubra* seeds determined by the Folin–Ciocalteu method, as the content of individual phenolic compounds was the subject of our previous research [10]. The phenolic compounds’ composition will not be discussed in detail here; nevertheless, the evaluation of the total phenolics content could not be neglected and excluded from the scope of this paper, since it would not give a complete overview regarding the abundance of components present in these valuable plants. Indeed, the health-promoting properties of acorns are due to the synergism of all the components that are available to the body after consumption. It is worth emphasizing that our previous study [10] on the effect of roasting conditions on the profile of phenolic compounds in northern red acorn seeds using ultra-high performance liquid chromatography coupled with diode array detection and high-resolution electrospray ionization mass spectrometry (UHPLC–DAD/ESI–HRMS/MS) showed that the predominant phenolic compounds in unroasted *Q. rubra* seeds were dimeric *m*-DOG type ellagitannins (rugosin E and oenothein B isomers) and phenolic glycosides (cretanin), followed by gallotannins (tetragalloylglucose and pentagalloylglucose). It was also demonstrated that, during roasting, the composition of phenolic compounds showed great qualitative and quantitative variations, depending on the roasting process parameters used.

The unroasted seeds contained significantly higher amounts of ellagitannins, gallotannins and phenolic glycosides in comparison with the roasted samples. On the other hand, the roasting process significantly increased the amounts of lower molecular weight gallotannins (i.e., mono- di-, and trigalloylglucose isomers), gallic acid, and ellagic acid and its derivatives compared to their initial values observed in unroasted seeds [10]. Based on these results, it can be concluded that roasting of *Q. rubra* seeds at 135 °C for 60 min was more favorable for preserving the highest amounts of bioactive ellagitannins, gallotannins and phenolic glycosides, while thermal processing at higher temperatures (180 and 200 °C) contributes to the formation of higher amounts of more bioavailable phenolic compounds, such as gallic acid, ellagic acid and its derivatives [10]. Similarly, Rakić et al. (2007) [2] noticed that the concentration of tannins decreased during heat treatment of *Q. robur* and *Q. cerris* kernels, while the level of gallic acid increased after thermal processing. Coelho et al. (2018) [5] also reported that roasting of *Q. suber* and *Q. ilex* cotyledons at 225–230 °C for 15 min affected their phenolic content. These results, however, slightly differed from those obtained by Rakić et al. (2007) [2], and revealed that the concentration of epicatechin gallate and gallic acid decreased after heat treatment, but the level of ellagic acid increased.

The abovementioned findings could be attributed to the decomposition of gallotannins, resulting in the formation of lower molecular weight gallotannins and gallic acid, and the release of compounds bounded to the cell wall macromolecules, including polysaccharides and proteins, during roasting of seeds [22,23,24,25,26]. In addition, thermal processing of plant materials causes the decomposition of ellagitannins, resulting in the formation of ellagic acid and other low molecular weight derivatives, such as valoneic acid dilactone or decarboxylated valoneic acid dilactone [27]. Recent studies have shown that the thermal stability of hydrolysable tannins may be affected by various factors, such as their chemical structure and the composition of the food matrix [2,25,26]. Taking into account that many competing reactions occur simultaneously during thermal treatment, these discrepancies in the results can be explained not only by variations in the composition of these phytochemicals in different *Quercus* species, but also by the experimental conditions used, especially the temperature and duration. For this reason, several authors may have obtained different results during the thermal processing of seeds of different oak types.

The slight decrease in total phenolics content of *Q. rubra* seeds observed in the present study despite large changes in the composition of individual phenolic compounds [10] can be also explained by the reactivity of the Folin–Ciocalteu reagent towards various compound classes [28]. The Folin–Ciocalteu test is an electron transfer-based assay that determines the reducing capacity of a solution expressed as phenolics content. This method is often used to determine the total phenolics content; however, the Folin–Ciocalteu reagent is not specific only to phenolic compounds. Therefore, other non-phenolic reducing agents, such as ascorbic acid, reducing sugars, amino acids, peptides, aromatic amines, organic acids and Maillard reaction products may react with the Folin–Ciocalteu reagent and interfere with the determination results [28]. Thus, the Folin–Ciocalteu test can determine not only phenolic compounds, but also other substances with reducing capacity. These substances are also very relevant due to displaying antioxidant activity. The findings of this study suggest that the formation of new antioxidant compounds, such as gallic acid, ellagic acid and its derivatives, can compensate for the degradation of naturally occurring phenolic compounds, which are potent antioxidants. In addition, the Maillard reactions occurring during the roasting of acorn seeds, especially at higher temperatures, can contribute to the formation of high-molecular-weight melanoidins, which are able to donate electrons [29].

Therefore, it can be concluded that roasting generally causes significant changes in the chemical composition of *Q. rubra* seeds, and the transformations of phenolic compounds occurring during heat treatment depend both on their chemical structure, which affects the susceptibility to oxidation and thermal stability of these compounds, and on the conditions of treatment (temperature and time). It is worth noting that the difference in the concentration of phenolic compounds in unroasted and roasted *Q. rubra* samples may result in differences in their biological activities and health-promoting properties.

#### 2.1.2. Effect of Roasting Conditions on Melanoidins

The results of the present study confirmed the possibility of the formation of high-molecular-weight, brown-colored Maillard reaction products (melanoidins) in acorn seeds during thermal processing. The concentration of melanoidins in the studied *Q. rubra* samples was determined by dialysis.

**Figure 2:**
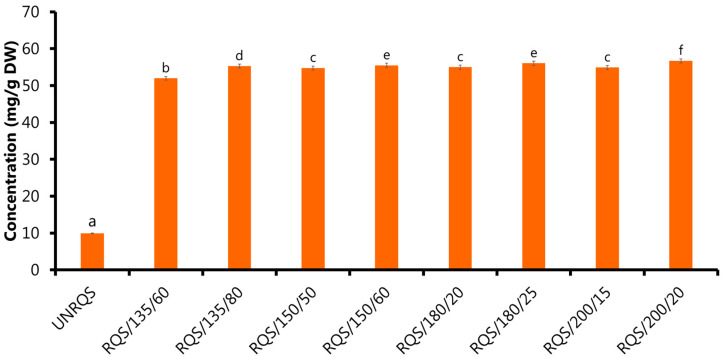
Content of melanoidins in unroasted and roasted *Q. rubra* seeds. Results are shown as the mean ± standard deviation of three replications (*n* = 3). Bars that share the same superscript letter (a–f) are not significantly different from each other (Tukey’s HSD test, *p* < 0.05).

The results showed significant differences (*p* < 0.05) in the high-molecular-weight, Maillard reaction products content of *Q. rubra* seeds subjected to roasting under different temperature and time conditions (Figure 2). It was observed that the content of these polymerized compounds in the roasted acorn samples increased significantly (*p* < 0.05) compared to the unroasted ones. Therefore, their concentration in roasted *Q. rubra* seeds was significantly higher than in unroasted samples (9.92 mg/g DW) and ranged from 51.96 to 56.69 mg/g DW. The greatest increase in the levels of melanoidins was recorded for samples heat-treated at 200 °C for 20 min, while the smallest changes were observed for these roasted at 135 °C for 60 min. To the best of our knowledge this is the first study to determine the content of melanoidins isolated by dialysis in thermally processed *Quercus* spp. seeds. Interestingly, these findings are in line with the literature as high temperature during the roasting of coffee or cocoa beans results in the increase levels of polymerized Maillard reaction products [30,31,32]. Due to the fact that acorn seeds contain proteins and carbohydrates [1,3], the reactions between reducing sugars and amino acids can occur during roasting, leading to the formation of various intermediate and end products of the Maillard reactions. In addition, some authors reported that oxidation and condensation of phenolic compounds could promote the production of melanoidins in heat treated foods [33,34]. Transformations of phenolic compounds accelerates polymerization reactions between oxidized phenolic compounds, polysaccharides, proteins and low molecular weight Maillard reaction products to form more complex structures [35,36].

One might presume an analogy that the phenolic compounds, especially phenolic acids, can be incorporated in the melanoidin structures during roasting, as in the case of coffee or cocoa beans through covalent and non-covalent interactions [30,37].

It should be noted that the presence of melanoidins in the roasted *Q. rubra* seeds is likely to increase biological value of this promising plant, as these compounds can contribute not only to the color and taste of heat-treated foods, but also have many health-promoting properties [17,34]. Melanoidins are bioactive compounds that have raised increasing interest in recent years, mostly due to their potent antioxidant, anti-inflammatory, antimicrobial, antihypertensive and chemopreventive properties [34,38,39]. These compounds can also act as dietary fiber in the gastrointestinal tract and promote the growth of beneficial gut bacteria [35,39]. Considering the beneficial biological properties of melanoidins, it is expected that products containing these compounds may be considered as promising food ingredients with the potential to improve health or reduce the risk of various diseases.

### 2.2. Effect of Roasting Conditions on Antioxidant Properties of Q. rubra Samples

In the present study, the antioxidant properties of unroasted and roasted, at different temperatures and times, *Q. rubra* seeds were analyzed by four different in vitro spectrophotometric assays, such as 2,2-diphenyl-1-picrylhydrazyl (DPPH) free radical-scavenging ability, ferric-reducing antioxidant power (FRAP) and ferrous ion chelating activity (Table 1). It was demonstrated that the roasting process parameters significantly (*p* < 0.05) affected the DPPH radical scavenging capacity of *Q. rubra* samples.

The DPPH test is widely used to assess the ability of compounds in food extracts to act as free radical scavengers or hydrogen donors, and thus their antioxidant capacity [40]. The unroasted seeds exhibited the highest DPPH radical scavenging capacity (5182.40 μM TE/g DW). It was noticed that the DPPH scavenging ability of *Q. rubra* samples significantly (*p* < 0.05) decreased during roasting and, depending on the processing conditions, varied from 3144.22 to 3943.94 μM TE/g DW. The most pronounced decrease in the DPPH capacity was observed upon roasting at 150 °C (by 36–39% of the initial value), regardless of the roasting time. The lowest reduction of the DPPH scavenging ability, as compared with unroasted samples, was observed when roasting was conducted at 200 °C for 20 min (by 24% of the initial value). The observed differences may be attributed to the complexity of changes in the chemical composition of acorn seeds occurring during roasting under different conditions. 

A noticeable reduction in the free radical scavenging capacity of roasted samples can be attributed to the degradation of hydrolysable tannins, especially ellagitannins. Importantly, the results of our previous research indicate that the unroasted *Q. rubra* seeds are characterized by a high content of phenolic compounds, including ellagitannins and gallotannins [10]. It is well established that these natural antioxidants are recognized as potential anti-inflammatory, anticancer, immunomodulatory, and antimicrobial agents [22]. However, it is well known that the DPPH radical scavenging capacity is related to the nature of phenolics and other compounds in the extracts and depends on their molecular structure, degree of hydroxylation, positions of hydroxyl groups in the structure, the availability of phenolic hydrogens and the ability to stabilize the resulting phenoxyl radicals by hydrogen donation or by extended electron delocalization [41]. Due to the presence of more phenolic hydroxyls per molecule of ellagitannins, the radical scavenging capacity of these complex compounds is higher than that of their low molecular weight degradation products, such as ellagic acid [42]. On the other hand, during the roasting, as a result of the reaction between reducing sugars and amino acids, Maillard reaction products can be formed, including melanoidins. These compounds are characterized by high free radical scavenging activity due to the presence of a number of active groups (OH or NH_2_), such as phenolic compounds, quinones, and low molecular weight Maillard reaction products, in their structure [43,44]. Therefore, the increase in temperature and time of thermal treatment, despite the greater degradation of ellagitannins and other high molecular weight phenolics, may have contributed to an increase in the free radical scavenging capacity of these samples compared to those roasted under less drastic conditions. In accordance with the results of the present study, Coelho et al. (2018) [5] also showed that the antioxidant capacity of roasted *Q. ilex* and *Q. suber* acorns (at 225–230 °C for 15 min) decreased compared to that of their unroasted counterparts. However, according to Rakić et al. (2007) [2] the DPPH radical scavenging activity of thermally treated *Q. robur* and *Q. cerris* kernels was higher than that of untreated ones.

The results of the present study revealed that thermal processing at temperatures between 135 and 200 °C generally caused a decrease in the ferric-reducing antioxidant activity of almost all *Q. rubra* samples (Table 1). Interestingly, the seeds roasted at 180 °C for 25 min had the highest FRAP value (1773.83 μM Fe(II)/g DW), which was even slightly higher compared to the initial value of the unroasted samples. Nevertheless, thermal treatment under the other roasting conditions tested in this study resulted in a decrease in the ferric-reducing antioxidant power. The most significant reduction in FRAP value (by 31% of the initial value) was observed when the seeds of *Q. rubra* were roasted at 150 °C for 60 min (1179.78 to μM Fe(II)/g DW). The FRAP assay is commonly used to measure the reducing potential based on the electron donating ability of phenolic compounds present in food extracts [41]. Hence, the decrease in the ferric-reducing antioxidant activity of seeds roasted at lower temperatures was mostly due to the degradation of ellagitannins and phenolic glycosides. On the contrary, only a slight decrease or increase in the reducing power of acorn seeds roasted at higher temperatures can be associated with the formation of new substances such as reductones, melanoidins and condensed phenolic compounds, which are capable of donating electrons or terminating radical chain reactions [43,44]. Slightly different results were obtained by Rakić et al. (2007) [2], who found that *Q. robur* and *Q. cerris* kernels thermally treated at 200 °C for 20 min showed higher ferric-reducing antioxidant power than untreated samples. The discrepancies between the results presented by different authors may be attributed to different roasting methods, different extraction procedures or diverse species of *Quercus* fruits.

In the present study, it was also observed that both the unroasted and roasted *Q. rubra* seeds possess strong ability to chelate ferrous ions (Table 1). These results indicate the great potential of acorn seeds as a functional bioactive ingredient since the ferrous ions are highly reactive and can catalyze the generation of potentially toxic reactive oxygen species (ROS) by the Fenton reaction, and accelerate lipid peroxidation by decomposing lipid hydroperoxides into peroxyl and alkoxyl radicals [40]. The ferrous ion chelating ability of *Q. rubra* samples decreased significantly (*p* < 0.05) with thermal processing (Table 1). The losses due to roasting ranged from 10 to 20% of the initial ability to chelate ferrous ions. The highest ferrous ion chelating activity was observed in unroasted seeds (32.40 mg EDTA/g DW), followed by those roasted at lowest temperatures and shorter time (29.23 mg EDTA/g DW). Similar to the FRAP power and DPPH scavenging capacity, the seeds roasted at 150 °C for 60 min showed the lowest ability to chelate ferrous ions (26.00 mg EDTA/g DW). The observed phenomenon may probably be attributed to the greatest degradation of ellagitannins and phenolic glycosides observed when seeds were roasted under these conditions. Among the roasted samples, the highest ferrous ion chelating activity (29.23 mg EDTA/g DW) was exhibited by the seeds roasted at 135 °C for 60 min, while the lowest ability was showed by those thermally treated at 150 °C for 60 min.

These differences can be explained by changes in the composition of the oak samples occurring during thermal processing, which are strongly determined by the process conditions. The results of the present study indicate that roasting significantly (*p* < 0.05) affected the chemical composition of *Q. rubra* seeds, with the effects being dependent on the processing conditions (Table S1). The moisture, crude protein and total fat content of acorn seeds gradually decreased as the roasting temperature increased from 135 to 200 °C. In contrast, thermal treatment of *Q. rubra* seeds caused an increase in ash and total carbohydrate content. It is possible that the elimination of water during roasting is responsible for the subsequent increase in ash and total carbohydrate content. However, changes in the carbohydrate content of roasted acorn seeds may also be caused by other factors. In this study, carbohydrate content was calculated by subtracting the total weight of moisture, crude protein, total fat and ash from the total weight of the sample. Therefore, the increase in carbohydrate content observed after roasting may be attributed to both the degradation of proteins and lipids, the formation of new compounds through the Maillard reactions and the release of cell wall-bound compounds during heat treatment [16,18,29,30,31,32].

These variations imply that thermal processing may affect the reducing capacity, free radical scavenging activity and ferrous ion chelating ability of *Quercus* seeds in different ways depending on the varietal differences of acorn seeds, the content of phenolics and melanoidins therein, and processing conditions used.

### 2.3. Effect of Roasting Conditions on Color of Q. rubra Samples

Color is a key food quality parameter that affects consumer preference of the final product. As can be seen in Table 2, total color difference (ΔE) of roasted *Q. rubra* seeds compared to unroasted samples increased significantly with the increase of temperature and time of roasting and varied from 21.00 to 34.75.

Hence, the color differences between the unroasted and heat-treated samples were obvious to the human eye [45]. In this study, it was observed that after roasting the L* values decreased significantly (*p* < 0.05) from 80.30 to a range of 47.64–62.79. On the other hand, the a* and b* values increased during heat treatments of studied samples from 1.97 to 8.15–10.44 and from 16.71 to 25.02–27.65, respectively. Similarly, compared to raw samples, the browning intensity of roasted *Q. rubra* seeds significantly increased (*p* < 0.05) with increasing temperature and roasting time (Table 2). The greatest changes in the CIE L*a*b* color parameters and browning index (BI) of studied samples were caused by roasting at 200 °C for 20 min, while the smallest modification was observed after thermal processing at 135 °C for 60 min. Similar results of color change were also reported by other authors [3], who noted that the browning of the acorn seeds increases with the progression of caramelization and Maillard reactions. These results are also consistent with the findings of Sun et al. (2022) [18], who showed that a decrease in the lightness (L*) and an increase in the redness (a*) indicate greater browning of the heat treatment of food, and these changes may be related to the formation of complex melanoidins.

### 2.4. Effect of Roasting Conditions on Taste of Q. rubra Samples

In addition to the health-promoting effects, phenolic compounds and Maillard reaction products are closely associated with the organoleptic properties of the plant-based foods. It is well known that ellagitannins, gallotannins, gallic acid, catechins and procyanidins are compounds directly related to the perception of astringency and bitter taste of pomegranate, nuts, red grapes, red wine, and dark chocolate [46,47]. One of the most important factors influencing consumer’s food acceptance and choices is its sensory appeal. The high amounts of tannins in most food products cause undesirable taste sensations, and efforts are being made to reduce their bitter taste. Therefore, the current study intended also to determine the influence of roasting conditions on the sensory properties of *Q. rubra* seeds. The taste quality (distinguished between different tastes) and its intensity of brews made from the unroasted and roasted *Q. rubra* samples were evaluated using an e-tongue with seven cross-selective sensors. The number of studies using electronic sensors, such as electronic tongues and noses, to test the sensory properties of food has increased recently. This is because analysis using an electronic sensor is non-destructive, allows for rapid taste analysis, and shows similarity to the results of human sensory test [48,49]. Based on the e-tongue analysis, differentiation of samples varying in flavors is possible. All seven sensor responses generated for each taste (bitter, sour, sweet, salty, umami, metallic and spicy) and for each sample were combined and transformed into sensory scores, which were used to describe the taste characteristics of the sample.

**Figure 3:**
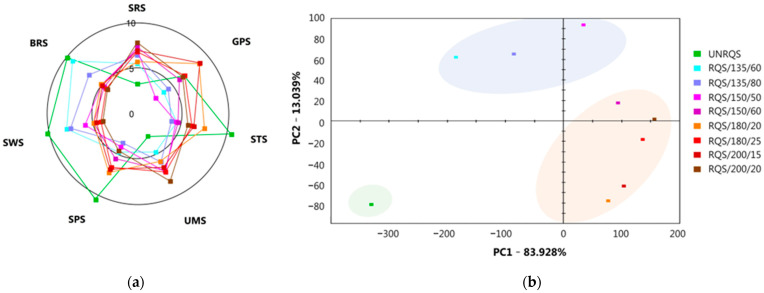
Radar chart representing taste scores of bars (**a**) and PCA graph showing the relationship among *Q. rubra* samples and tastes (**b**). SRS, sourness; GPS, metallic; STS, saltiness; SPS, spiciness; UMS, umami; SWS, sweetness; BRS, bitterness.

The results of this study indicated that the roasting temperature and time significantly affect the sensory attributes of the studied *Q. rubra* samples (Figure 3). It was observed that the unroasted samples showed the highest bitter and sweet tastes, which gradually decreased with increasing temperature or roasting time. However, the roasted samples revealed some similarity in their taste profiles but exhibited lower intensities of most taste characteristics than that of the unroasted seeds. In terms of bitter taste of roasted samples, the highest and lowest values of 9.2 and 4.2 were observed for the seeds thermally treated at 135 °C for 60 min and at 200 °C for 20 min, respectively. A similar trend was observed in the case of sweet taste. The lowest value (3.90) was found for the samples roasted at highest temperature and longer time, while the highest (8.0) was found in those thermally treated at lowest temperature and shorter time. In terms of salty taste, the highest (7.6) and lowest (3.9) values were observed after roasting at 180 °C for 20 min and at 135 °C for 80 min, respectively. The temperature and time of roasting also affected two other primary sensory characteristics (i.e., sour and umami) of *Q. rubra* samples. The intensities of these tastes increased with roasting temperature and time, with the taste values ranking from 5.4 to 7.8 and from 3.3 to 8.3, respectively.

The discrepancies in the behavior of different tastes during thermal processing may be related to changes in the chemical composition of *Q. rubra* seeds. In our previous study, it was found that the content of hydrolysable tannins (ellagitannins and gallotannins) and phenolic glycosides decreased significantly after thermal processing [10], and, accordingly, in the present study the total phenolics content and bitterness of roasted samples also decreased (Figure 3a). It has already been proven that the presence of some of these phenolic compounds in foods has been associated with sensory and health promoting properties [15]. The bitter taste of *Quercus* seeds is mainly related to their high tannin content [1]. As described by other authors, the seeds of northern red oaks are considered as more bitter and tannin-rich than other oaks [50]. Nevertheless, the chemical composition of the oak fruits depends not only on the species, but also on the other factors, such as genetic variation within the same species, growth geographical area, the environment conditions, harvest time and maturity and the variability post-harvest processing [7,8,51]. According to Łuczaj et al. (2014) [50], the total tannin content was lower in the acorns of *Q. rubra* than in samples of *Q. petraea* and *Q. robur*, while the total and non-tannin phenolics concentrations in northern red oaks were higher than other oak species. In accordance with previous papers, the results presented in this study demonstrate that not only tannins, but also other phenolic compounds, especially phenolic glycosides, primarily, contribute to the bitterness of unroasted *Q. rubra* seeds. Therefore, the higher content of ellagitannins and phenolic glycosides in samples roasted at 135 °C for 60 min may be responsible for their more bitter taste compared with other thermally treated samples. In addition, regarding the sweet taste of studied *Q. rubra* seeds, with increasing temperature and roasting time, thermal degradation of some sugars occurs and thus sweetness decreases [49]. The results obtained from this study indicate that roasting conditions such as temperature and time greatly affect the content of individual and total free sugars (a sum of fructose, glucose, and sucrose) in studied *Q. rubra* samples (Table 3). It was found that the total sugar and sucrose contents significantly decreased (*p* < 0.05) with an increase of temperature and time of the heating medium, while the level of fructose noticeably increased compared to the unroasted seeds. In turn, the concentration of glucose increases or decreases depending on the roasting conditions. These results may be attributed to the hydrolysis of sucrose to fructose and glucose at high temperatures. Further thermal dehydration of these reducing sugars can contribute to the generation of acid precursors, while their polymerization with amino acids, proteins or other reactive intermediates can generate the high molecular weight Maillard reaction products, especially melanoidins [52]. 

Therefore, the reduction of the total free sugars and phenolic compounds content could promote melanoidins formation (Figure 1 and Figure 2 and Table 3). In addition, thermal degradation of sugars, especially sucrose, could contribute to the formation of carboxylic acids, including acetic acid, thereby increasing the acidity of the roasted samples [53]. These findings are consistent with the results of the present study, which showed that thermal treatment at temperatures between 135 and 200 °C generally caused a decrease in the pH value of *Q. rubra* samples (Table 3). Other taste characteristics of studied samples can also be affected by the thermal processing. This is because chemical composition of *Q. rubra* seeds undergoes significant modification during roasting, and these changes can contribute to an increase or decrease in the intensity of different types of tastes (bitter, sour, sweet, salty and umami).

The taste characteristics of unroasted and roasted *Q. rubra* seeds were discriminated using principal component analysis (PCA) and separation of samples along the two principal components (PC1 and PC2) based on roasting conditions was evident (Figure 3b). The PCA plot showed that about 97% of the variation was explained in the first two PCs, with 83.928% explained by PC1 and 13.039% explained by PC2. These differences seem to derive from the different composition of water-soluble taste-active compounds present in northern red oak samples. The unroasted *Q. rubra* seeds were clearly separated from roasted ones, indicating the different taste profiles of these samples.

The unroasted and thermally treated samples were separated along PC1 and PC2. The patterns of the taste components of the water extracts were shown to change depending on the roasting conditions. Samples roasted at lowest temperature (135 °C), regardless of roasting time, were located in the negative (−) direction based on PC2 and in the positive (+) direction based on PC1, while those thermally treated at higher temperatures (150 and 180 °C) were located in the positive (+) direction based on both PC1 and PC2. The changes in the taste components of samples roasted at higher temperatures (180 and 200 °C) were more significant than these thermally treated at lower temperatures. In addition, samples roasted at the same temperatures (150–200 °C) but different times also showed a difference in their taste profiles. This separation occurred along PC2 and resulted from differences in the intensity of sweetness and sourness of roasted samples. This behavior may be explained by improved diffusion of heat into the seeds as a result of longer heat treatment, which leads to intensive chemical composition changes due to hydrolysis, oxidation or condensation reactions of taste-active compounds, including phenolic compounds, sugars, amino acids, peptides, Maillard reaction products, organic acids and inorganic ions [46,47,54].

## 3. Materials and Methods

### 3.1. Chemical Reagents and Materials

Gallic acid, acetonitrile of HPLC grade (≥99.9%), 6-hydroxy-2,5,7,8-tetramethylchroman-2-carboxylic acid (Trolox), 2,2-diphenyl-1-picrylhydrazyl (DPPH), 2,4,6-tri(2-pyridyl)-s-triazine (TPTZ), sodium acetate, ferric chloride hexahydrate, ferrozine, ammonium acetate and disodium ethylenediaminetetraacetate dihydrate were all obtained from Sigma-Aldrich (St. Louis, MO, USA). Hydrochloric acid (HCl), sodium chloride (NaCl) and monosodium glutamate (MSG) solutions were purchased from Chem-Lab NV (Zedelgem, Belgium). Water was purified by a Milli-Q water purification system (Millipore Corp., Bedford, MA, USA). All other chemicals were of analytical grade and reagents were prepared according to standard analytical procedures.

### 3.2. Plant Materials

**Figure 4:**
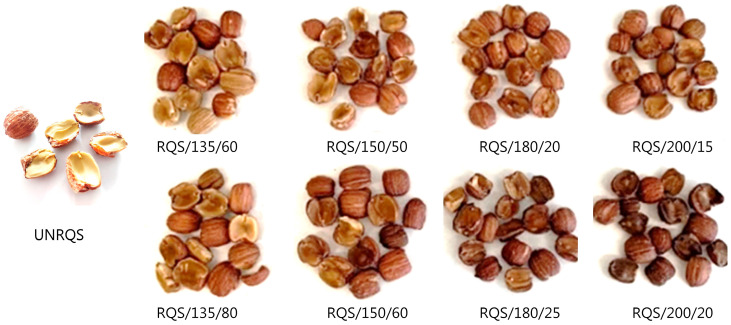
Representative images of unroasted and roasted *Q. rubra* (syn. *Q. borealis* F. Michx) seeds; UNRQS, the unroasted seeds; RQS/135/60, seeds roasted at 135 °C/60 min, RQS/135/80, seeds roasted at 135 °C/80 min; RQS/150/50, seeds roasted at 150 °C/50 min; RQS/150/60, seeds roasted at 150 °C/60 min; RQS/180/20, seeds roasted at 180 °C/20 min; RQS/180/25, seeds roasted at 180 °C/25 min; RQS/200/15, seeds roasted at 200 °C/15 min; RQS/200/20, seeds roasted at 200 °C/20 min.

The fully ripe acorns of *Q. rubra* (syn. *Q. borealis* F. Michx) were collected in October 2021 from forest located in southern Poland (GPS location: 50°04′18.5″ N, 20°35′09.0″ E). After harvesting, the fruits were subjected to cleaning and sun-drying, manually deshelled, and then immediately used in further experiments. Seeds were roasted in batches of 50 ± 0.1 g in a convective roaster CBR-101 (Gene Cafe, France) under the following temperature and time conditions: 135 °C for 60 and 80 min, 150 °C for 50 and 60 min, 180 °C for 20 and 25 min, and at 200 °C for 15 and 20 min [10]. After roasting, seeds were cooled to the temperature about 20 °C using cold air, vacuum-packed in plastic bags and stored at −20 °C until further analysis. All roasting experiments were performed in three replicates. The roasting conditions used in this study were selected based on the results of previous studies [2,3,10]. Prior to analysis, both unroasted and roasted seeds (Figure 4) were ground to obtain a fine powder and passed through a 32-mesh sieve (0.50 mm). The moisture content of each sample was determined in triplicate by drying in an oven (102 ± 1 °C) until a constant weight.

### 3.3. Phenolic Compounds Determination

Total phenolic contents of the extracts of *Q. rubra* samples were determined using the Folin–Ciocalteu method, as described by Oracz and Żyżelewicz (2019) [43]. The extracts from tested samples were prepared according to the method described by Oracz et al. (2022) [10]. Briefly, one gram of each sample was extracted with 50 mL of 70% aqueous methanol by sonication for 30 min at 45 °C. The ultrasonic frequency was 37 kHz, and the power was 150 W. The extracts were centrifuged at 4800× *g* for 10 min at 4 °C, and the residue was re-extracted twice under the same conditions. The supernatants of the three extractions were pooled and filtered through Whatman no. 4 filter paper before being subjected to further analysis. All measurements were carried out in triplicate. The total phenolics content was calculated from the calibration curve prepared with the use of gallic acid as a standard (range 2–105 mg/L, y = 0.0125x, R^2^ = 0.9984). The results were expressed as milligrams of gallic acid equivalents (GAE) per gram of DW (mg GAE/g DW).

### 3.4. Melanoidins Determination

The melanoidins content was determined according to the method proposed previously by Oracz et al. (2019) [30], with some modifications. Briefly, samples of *Q. rubra* samples (5 g) were extracted twice with 50 mL of distilled water at 90 °C for 20 min in an orbital shaker (100× *g*). After cooling to room temperature, the extracts were centrifuged at 4800× *g* for 10 min at 20 °C. The supernatants were pooled, filtered through Whatman no. 4 filter paper and transferred to dialysis tubes (MW cutoff >12.4 kDa, Sigma-Aldrich, Saint Louis, MO, USA). Dialysis was carried out for the first day under running tap water and for subsequent days against 2000 mL of distilled water at 4 °C with constant stirring. After dialysis, the retentate containing melanoidins was frozen at −20 °C and lyophilized (−50 °C, 0.9 mbar) using a DELTA 1-24LSC Christ freeze drier (Martin Christ, Osterode am Harz, Germany). The lyophilized samples were weighed and melanoidins content was calculated. All experiments were carried out in triplicate. Results of the melanoidins determination were expressed as milligrams per gram of DW (mg/g DW).

Melanoidins concentration was calculated using the following equation:

Melanoidins content (mg/g DW) = X_1_/X_2_ (1)

where X_1_ is the weight (mg) of lyophilized melanoidins fraction and X_2_ is the weight (g) of acorn samples’ dry weight.

### 3.5. Antioxidant Activity Analysis

#### 3.5.1. Determination of Free Radical-Scavenging Capacity

The free radical-scavenging activity of the extracts of *Q. rubra* samples prepared as described above in Section 3.3. was determined by the DPPH assay as previously described by Oracz and Żyżelewicz (2019) [43]. All experiments were carried out in triplicate. The DPPH radical scavenging activity was calculated from the calibration curve prepared using Trolox as a standard (range 0.02–0.25 μmol/L, y = 5.1805x, R^2^ = 0.9995). The results were expressed as µM Trolox equivalents per gram of DW (μM TE/g DW).

#### 3.5.2. Determination of Ferric Reducing Antioxidant Power

The ferric reducing antioxidant power (FRAP) of the extracts of *Q. rubra* samples prepared as described above in Section 3.3. was determined according to the protocol described by Oracz and Żyżelewicz (2019) [43]. A calibration curve was constructed using ferrous sulphate heptahydrate (FeSO_4_·7H_2_O): range 0.01–0.20 μmol/L, y = 4.9799x, R^2^ = 0.9989. All experiments were carried out in triplicate. Results were expressed as µM Fe(II) equivalents per gram of DW (μM Fe(II)/g DW).

#### 3.5.3. Determination of Ferrous Ion Chelating Activity

The chelation of Fe(II) ions by the extracts of *Q. rubra* samples prepared as described above in Section 3.3. was determined as described by Oracz and Żyżelewicz (2019) [43]. A calibration curve was constructed using disodium ethylenediaminetetraacetate dihydrate (EDTA): range 2.4–80 mg/L, y = 1.5571x, R^2^ = 0.9945. All experiments were carried out in triplicate. Results were expressed as milligrams of EDTA equivalents per gram of DW (mg EDTA/g DW).

### 3.6. CIE L*a*b* Color and Browning Index Determination

Color evaluation of *Q. rubra* samples was performed using a Konica Minolta CR-400 Chroma meter with Spectra Magic NX 1.3 software (Konica Minolta, Osaka, Japan), and the results were expressed in accordance with the CIE L*a*b* system with CIE Standard Illuminant D65 as a light source. The CIE (Commission International de l’Eclairage) color values were L* (lightness), a* (redness) and b* (yellowness) were determined. All measurements were performed in triplicate.

The Browning index (BI) was calculated using the following equations [55]:

BI = [100 (X − 0.31)]/0.17(2)

where X is the chromaticity coordinate calculated from L*a*b* values, as X = (a* + 1.75 L*)/(5.645 L* + a* − 0.3012 b*).

### 3.7. Determination of pH

The pH values of *Q. rubra* samples were determined according to the methodology proposed by the Purabdolah et al. (2020) [56], with some modifications. For pH determination, 2.5 g of ground sample was extracted with distilled water (100 mL) for 20 min at 60 °C using shaking water bath. The resulting aqueous extracts were centrifuged (4800× *g* for 10 min at 20 °C) and then filtered through Whatman no. 4 filter paper. The pH of the samples was determined using an ELMETRON CP-505 digital pH meter with an EPP-1 electrode (Zabrze, Poland). All measurements were performed in triplicate.

### 3.8. Proximate Analysis

The protein content of acorn seeds was determined using the Kjeldahl method described in AOAC 970.22 [57], using a nitrogen to protein conversion factor of 6.25. Total fat content was determined gravimetrically after acid hydrolysis and Soxhlet extraction with petroleum ether, according to AOAC 963.15 [57]. Ash was determined using a gravimetric method, according to AOAC 942.05 [58] by burning samples at 650 °C to constant weight. Total carbohydrates were determined using the differential calculation method.

### 3.9. Determination of Free Sugars by HPLC

The free sugars’ (glucose, sucrose and fructose) composition of *Q. rubra* samples was analyzed according to the method reported by Oracz and Nebesny (2019) [59], with some modifications. The water extracts prepared as described above in Section 3.7. were passed through 0.45 μm nylon syringe filters and analyzed for the content of free sugars using a UHPLC+ Dionex UltiMate 3000 system (Thermo Fisher Scientific Inc., Waltham, MA, USA) equipped with a refractive index detector (Shimadzu, Japan) and an Asahipak NH2P-50 4E column (4.6 × 150 mm, 5.0 μm particle size; Shodex, Japan). Isocratic elution was carried out with acetonitrile/water (70/30, *v*/*v*) as the mobile phase. The flow rate and column temperature were set at 1.0 mL/min and 30 °C, respectively. Glucose, sucrose and fructose were identified by comparing their retention times with authentic standards. Quantification was carried out using an external standard method. Calibration curves were prepared from standard solutions of sugars in the following concentration ranges: 0.1–10 mg/mL for glucose (y = 0.7411x, R^2^ = 0.9984), 0.1–15 mg/L for sucrose (y = 0.9252x, R^2^ = 0.9976), and 0.1–10 mg/mL for fructose (y = 0.6939, R^2^ = 0.9995). All measurements were performed in triplicate. Results were expressed as mg/g DW.

### 3.10. Taste Evaluation by Electronic Tongue

Instrumental taste analysis of *Q. rubra* samples was carried out using the Alpha MOS ASTREE II electronic tongue (e-tongue) instrument (Alpha MOS., Toulouse, France), consisting of a 48 position autosampler for the automated analysis of a samples set in reproducible conditions (time, stirring), an array of liquid sensors (set #5, SRS-sourness, STS-saltiness, UMS-umami, SWS-sweetness, and BRS-bitterness, GPS-metallic and SPS-spiciness), and a reference electrode (Ag/AgCl) according to the methodology proposed by Kowalski et al. (2022) [60]. Aqueous extracts of unroasted and roasted acorn seeds were prepared prior to measurements as described above in Section 3.7, and transferred to 20 mL glass autosampler beakers. The analysis time was set at 120 s and data from the last 20 s were used for acquisition. After successful conditioning and testing of the e-tongue sensors, each aqueous extract from a sample of *Q. rubra* was analyzed five times. The sensors were rinsed with distilled water for 120 s after each sample measurement. Data obtained from the e-tongue were further analyzed using AlphaSoft software (Alpha MOS., Toulouse, France). The taste screening analysis was used to rank the samples according to taste attributes on a 0 to 12 intensity scale. The principal component analysis (PCA) was performed to discriminate the response signals from the seven sensors. Discrimination was achieved by comparing the distances and pattern discrimination indices between sample groups on a PCA score plot [60].

### 3.11. Statistical Analysis

The results of three independent experiments were expressed as the mean ± standard deviation (SD). The Statistica 13.0 software (StatSoft, Inc., Tulsa, OK, USA) was used for the statistical data analyses. The significant differences among tested characteristics (i.e., phenolic compounds, melanoidins, sugars, antioxidant properties, color, taste and pH of the *Q. rubra* samples were estimated by the one-way analysis of variance (ANOVA) followed by Tukey’s Honest Significant Difference (HSD) test. Differences were considered to be significant when *p* values were less than 0.05 (*p* < 0.05).

## 4. Conclusions

The results presented in this study clearly indicate that both unroasted and roasted seeds of *Q. rubra* can be considered as a valuable source of bioactive compounds with strong antioxidant properties. Northern red oak acorns can therefore potentially be used to produce a wholesome, healthy food rich in all essential nutrients, the consumption of which can reduce the risk of developing lifestyle diseases. In addition, the high content of phenolic compounds and melanoidins will make them and their preparations more stable and resistant to pests and pathogens, extend their expected storage time and improve processing ability without the undesirable necessity of stabilizers and preservatives usage. Thus, seeds of *Q. rubra* are practically free from cultivation, fertilization or pesticide use and can be considered as a relevant source of functional foods.

Finally, the results also showed that both unroasted and roasted seeds of *Q. rubra* can be used not only as a functional ingredient in the food industry but also potentially in the pharmaceutical and cosmetic ones.

## Figures and Tables

**Table 1:** Antioxidant properties of *Q. rubra* seeds before and after roasting.

Samples	DPPH (μM TE/g DW)	FRAP (μM Fe(II)/g DW)	Fe(II) Chelating Ability (mg EDTA/g DW)
UNRQS	5182.40 ± 3.98 ^g^	1703.76 ± 2.70 ^f^	32.40 ± 0.27 ^g^
RQS/135/60	3789.36 ± 3.12 ^d,e^	1646.61 ± 2.56 ^e^	29.23 ± 0.28 ^f^
RQS/135/80	3505.76 ± 2.98 ^c^	1286.08 ± 2.39 ^b^	28.09 ± 0.31 ^d,e^
RQS/150/50	3316.91 ± 4.09 ^b^	1365.33 ± 2.45 ^c^	26.48 ± 0.19 ^a,b^
RQS/150/60	3144.22 ± 3.72 ^a^	1179.78 ± 2.71 ^a^	26.00 ± 0.21 ^a^
RQS/180/20	3716.70 ± 3.19 ^d^	1500.64 ± 2.38 ^d^	28.58 ± 0.17 ^e,f^
RQS/180/25	3827.15 ± 3.45 ^e^	1773.83 ± 2.25 ^g^	26.98 ± 0.23 ^b,c^
RQS/200/15	3562.03 ± 2.87 ^c^	1488.90 ± 2.08 ^d^	28.79 ± 0.18 ^e,f^
RQS/200/20	3943.94 ± 3.09 ^f^	1472.40 ± 2.62 ^d^	27.71 ± 0.25 ^c,d^

Data are shown as mean ± standard deviation, *n* = 3, and values followed by different superscript letters (a–g) in the same column are significantly different (Tukey’s HSD test, *p* < 0.05); DPPH, free radical scavenging activity against 2,2-diphenyl-1-picrylhyl radicals; TE, Trolox equivalents; FRAP, the ferric reducing antioxidant power; Fe(II) chelating ability, ferrous ion chelating ability; EDTA, the ethylenediaminetetraacetic acid equivalents.

**Table 2:** CIE L*a*b* color parameters and browning index of *Q. rubra* seeds before and after roasting.

Samples	L*	a*	b*	ΔE	BI
UNRQS	80.30 ± 0.23 ^g^	1.97 ± 0.11 ^a^	16.71 ± 0.10 ^a^	-	3.82 ± 0.10 ^a^
RQS/135/60	62.79 ± 0.28 ^f^	8.15 ± 0.08 ^b^	26.51 ± 0.06 ^c^	21.00 ± 0.11 ^a^	13.44 ± 0.11 ^b^
RQS/135/80	58.51 ± 0.19 ^c^	9.36 ± 0.09 ^d^	27.50 ± 0.07 ^e^	25.42 ± 0.13 ^d^	16.03 ± 0.13 ^e^
RQS/150/50	57.97 ± 0.25 ^c^	9.41 ± 0.08 ^d^	27.41 ± 0.11 ^d,e^	25.86 ± 0.10 ^e^	16.22 ± 0.10 ^f^
RQS/150/60	53.34 ± 0.26 ^b^	10.38 ± 0.13 ^f^	27.20 ± 0.12 ^d^	30.13 ± 0.09 ^g^	18.82 ± 0.11 ^h^
RQS/180/20	59.41 ± 0.28 ^d^	9.33 ± 0.10 ^d^	27.65 ± 0.08 ^e^	24.70 ± 0.12 ^c^	15.78 ± 0.10 ^d^
RQS/180/25	53.83 ± 0.16 ^b^	10.23 ± 0.07 ^e^	27.19 ± 0.10 ^d^	29.65 ± 0.11 ^f^	18.46 ± 0.12 ^g^
RQS/200/15	61.26 ± 0.25 ^e^	8.60 ± 0.09 ^c^	26.78 ± 0.13 ^c^	22.53 ± 0.10 ^b^	14.33 ± 0.13 ^c^
RQS/200/20	47.64 ± 0.29 ^a^	10.44 ± 0.11 ^f^	25.02 ± 0.10 ^b^	34.75 ± 0.14 ^h^	20.65 ± 0.14 ^i^

Data are shown as mean ± standard deviation, *n* = 3, and values followed by different superscript letters (a–g) in the same column are significantly different (Tukey’s HSD test, *p* < 0.05); BI, browning index.

**Table 3:** Th pH and free sugars content in *Q. rubra* seeds before and after roasting.

Samples	pH	Free Sugars (mg/g DW)			
		Fructose	Glucose	Sucrose	Total
UNRQS	4.97 ± 0.03 ^f^	7.67 ± 0.06 ^a^	20.05 ± 0.07 ^c^	122.13 ± 0.09 ^g^	149.85 ± 0.12 ^g^
RQS/135/60	4.65 ± 0.04 ^d^	13.40 ± 0.07 ^f^	21.15 ± 0.06 ^e^	85.38 ± 0.08 ^f^	119.93 ± 0.14 ^f^
RQS/135/80	4.61 ± 0.02 ^c^	12.28 ± 0.04 ^e^	15.89 ± 0.09 ^a^	67.98 ± 0.07 ^c^	96.15 ± 0.13 ^b^
RQS/150/50	4.57 ± 0.05 ^b^	14.76 ± 0.03 ^h^	20.64 ± 0.07 ^d^	69.52 ± 0.04 ^d^	104.92 ± 0.16 ^c^
RQS/150/60	4.55 ± 0.03 ^b^	15.48 ± 0.05 ^i^	23.44 ± 0.04 ^f^	57.40 ± 0.05 ^b^	96.32 ± 0.15 ^b^
RQS/180/20	4.68 ± 0.07 ^e^	11.02 ± 0.06 ^c^	19.09 ± 0.05 ^b^	75.29 ± 0.08 ^e^	105.40 ± 0.10 ^c^
RQS/180/25	4.63 ± 0.04 ^c,d^	14.49 ± 0.05 ^g^	24.87 ± 0.06 ^g^	68.35 ± 0.09 ^c^	107.71 ± 0.14 ^e^
RQS/200/15	4.61 ± 0.03 ^c^	11.74 ± 0.07 ^d^	19.13 ± 0.09 ^b^	75.35 ± 0.07 ^e^	106.22 ± 0.12 ^d^
RQS/200/20	4.52 ± 0.05 ^a^	10.05 ± 0.04 ^b^	23.47 ± 0.08 ^f^	44.27 ± 0.06 ^a^	77.79 ± 0.13 ^a^

Data are shown as mean ± standard deviation of three replications (*n* = 3), and values followed by different superscript letters in the same column are significantly different (Tukey’s HSD test, *p* < 0.05); UNRQS, the untreated seeds; RQS/135/60, seeds roasted at 135 °C/60 min, RQS/135/80, seeds roasted at 135 °C/80 min; RQS/150/50, seeds roasted at 150 °C/50 min; RQS/150/60, seeds roasted at 150 °C/60 min; RQS/180/20, seeds roasted at 180 °C/20 min; RQS/180/25, seeds roasted at 180 °C/25 min; RQS/200/15, seeds roasted at 200 °C/15 min; RQS/200/20, seeds roasted at 200 °C/20 min.

## Data Availability

Data available on request.

## Supplementary Materials

The following supporting information can be downloaded at: https://www.mdpi.com/article/10.3390/molecules28052299/s1, Table S1: Proximate composition (%) of *Q. rubra* seeds before and after roasting.

## Sample Availability

Samples of phenolic compounds are available from the authors.
