# Supplementary material for: Bioactive Compounds, Antioxidant Activity and Sensory Properties of Northern Red Oak (Quercus rubra L., syn. Q. borealis F. Michx) Seeds Affected by Roasting Conditions
Source: Molecules. 2023 Mar 1;28(5):2299. doi: 10.3390/molecules28052299 (PMC10005602; doi:10.3390/molecules28052299)
Supplement: Supplementary file 1 [file molecules-28-02299-s001.zip › molecules-2191355-supplementary.pdf]

molecules

Supplementary Materials

Bioactive Compounds, Antioxidant Activity and Sensory Properties of Northern Red Oak (*Quercus rubra* L., syn. *Q. borealis* F. Michx) Seeds Affected by Roasting Conditions

Joanna Oracz *, Monika Prejzner, Joanna Grzelczyk, Gabriela Kowalska, Dorota Żyżelewicz

Institute of Food Technology and Analysis, Faculty of Biotechnology and Food Sciences, Lodz University of Technology, 2/22 Stefanowskiego Street, 90-537 Lodz, Poland

* Correspondence: joanna.oracz@p.lodz.pl; Tel.: +48 42 631 34 62, Fax: +48 42 631 32 43

Contents

Table S1. Proximate composition (%) of *Q. rubra* seeds before and after roasting.

Table S1. Proximate composition (%) of *Q. rubra* seeds before and after roasting.

Samples	Moisture	Crude protein	Fat	Ash	Carbohydrate
UNRQS	14.96 ± 0.10^{h}	5.53 ± 0.04^{d}	16.10 ± 0.19^{a}	1.90 ± 0.07^{a}	61.50 ± 0.39^{a}
RQS/135/60	8.05 ± 0.09^{g}	5.30 ± 0.05^{b}	14.53 ± 0.15^{d}	2.17 ± 0.05^{c}	69.95 ± 0.34^{b}
RQS/135/80	5.69 ± 0.07^{f}	5.20 ± 0.03^{a}	14.95 ± 0.16^{e}	2.24 ± 0.08^{e}	71.91 ± 0.34^{c}
RQS/150/50	3.67 ± 0.10^{e}	5.51 ± 0.06^{d}	13.99 ± 0.12^{b}	2.28 ± 0.07^{f}	74.56 ± 0.35^{d}
RQS/150/60	3.66 ± 0.09^{e}	5.43 ± 0.04^{c}	14.01 ± 0.09^{b,c}	2.31 ± 0.09^{g}	74.58 ± 0.32^{d}
RQS/180/20	3.59 ± 0.05^{d}	5.31 ± 0.05^{a}	14.47 ± 0.14^{d}	2.20 ± 0.05^{d}	74.43 ± 0.29^{d}
RQS/180/25	2.65 ± 0.06^{b}	5.18 ± 0.07^{a}	13.80 ± 0.16^{a}	2.32 ± 0.06^{g}	76.06 ± 0.35^{e}
RQS/200/15	3.50 ± 0.09^{c}	5.44 ± 0.05^{c}	14.17 ± 0.16^{c}	2.12 ± 0.07^{b}	74.77 ± 0.37^{d}
RQS/200/20	2.22 ± 0.07^{a}	5.39 ± 0.08^{c}	13.86 ± 0.15^{a,b}	2.41 ± 0.08^{h}	76.13 ± 0.38^{e}

Data are shown as mean ± standard deviation, n = 3, and values followed by different superscript letters (a–h) in the same column are significantly different (Tukey's HSD test, $p < 0.05$); UNRQS, the unroasted seeds; RQS/135/60, seeds roasted at 135 °C/60 min, RQS/135/80, seeds roasted at 135 °C/80 min; RQS/150/50, seeds roasted at 150 °C/50 min; RQS/150/60, seeds roasted at 150 °C/60 min; RQS/180/20, seeds roasted at 180 °C/20 min; RQS/180/25, seeds roasted at 180 °C/25 min; RQS/200/15, seeds roasted at 200 °C/15 min; RQS/200/20, seeds roasted at 200 °C/20 min.
